# An AnteOwl WR intravascular ultrasound-guided parallel wiring technique for chronic total occlusion of below-the-knee arteries

**DOI:** 10.1186/s42155-022-00294-2

**Published:** 2022-03-26

**Authors:** Naoki Hayakawa, Satoshi Kodera, Satoshi Hirano, Masataka Arakawa, Yasunori Inoguchi, Junji Kanda

**Affiliations:** 1 Department of Cardiovascular Medicine, Asahi General Hospital, I-1326, Chiba 289-2511 Asahi, Japan; 2 Department of Cardiovascular Medicine, The University of Tokyo Hospital, Tokyo, Japan

**Keywords:** Chronic total occlusion, Endovascular therapy, Intravascular ultrasound, Below-the knee

## Abstract

**Background:**

Although endovascular therapy is used to treat chronic limb-threatening ischemia, long chronic total occlusion (CTO) is still challenging to treat. Especially in patients with poor run-off below-the-knee (BTK) arteries, it is difficult to perform a retrograde approach, and even guidewire passage may be difficult.

**Case presentation:**

We treated two cases of chronic limb-threatening ischemia using our novel extreme antegrade guidewire crossing technique by AnteOwl WR intravascular ultrasound (IVUS)-guided parallel wiring to a BTK artery (EXCAVATOR technique). Case 1 was a 70-year-old man with ulceration of the right toe. The AnteOwl WR IVUS was intentionally advanced into the subintimal space of the posterior tibial artery, and the totally intraplaque route was advanced by IVUS-guided parallel wiring that was successfully passed from the lateral plantar aspect to the true lumen of the digital artery. Case 2 was a 76-year-old woman with rest pain and cyanosis of the right lower limb. Angiography showed total occlusion from the superficial femoral artery to BTK arteries. AnteOwl WR IVUS-guided parallel wiring was repeatedly performed until the distal true lumen of the peroneal artery was reached, and revascularization was successfully achieved via the antegrade approach alone.

**Conclusions:**

With its excellent crossable performance, good image quality, and high navigational ability within the CTO, the AnteOwl WR can be used to pass parallel wiring into the distal true lumen for BTK CTO.

## Background

Endovascular therapy (EVT) is now a widespread method of revascularization for patients with chronic limb-threatening ischemia (CLTI); however, it is often difficult to use EVT to treat long chronic total occlusion (CTO) in below-the-knee (BTK) lesions (Iida et al. 2017; Lyden et al., 2009). Although the success of EVT in such lesions has been improved by the introduction of retrograde approaches such as distal puncture, retrograde approaches are extremely difficult to use for lesions with poor distal target vessels (Schmidt et al. 2017). Furthermore, the presence of poor run-off vessels often makes it difficult to perform bypass surgery.

**Fig. 1:**
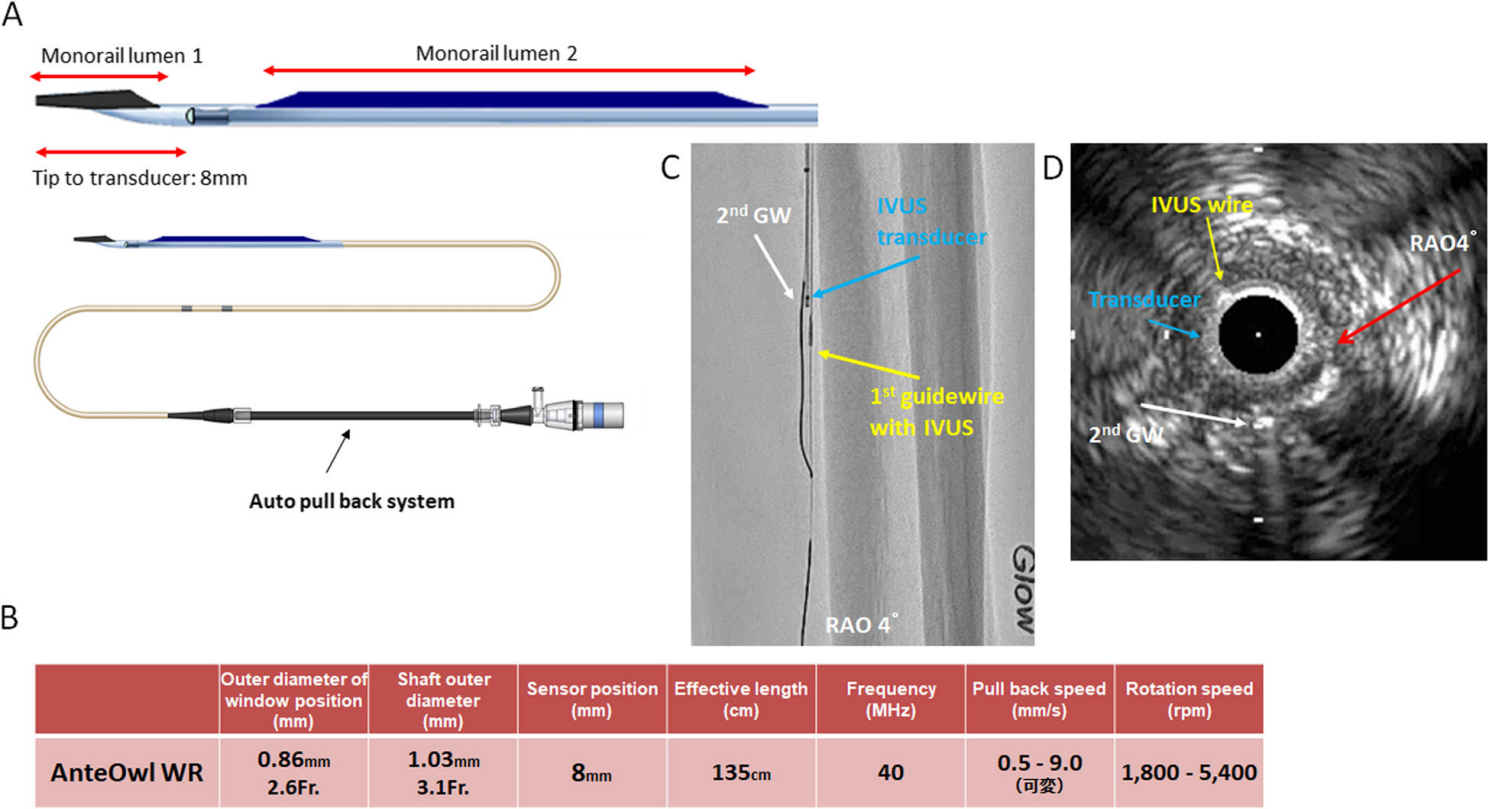
**A** Structure of the AnteOwl WR (AnteOwl) intravascular ultrasound (IVUS). **B** Specifications of the AnteOwl. **C** Angiographic images of the AnteOwl in a below-the-knee lesion in the right anterior oblique (RAO) 4° view. The 1st guidewire (yellow arrow) and AnteOwl IVUS transducer (blue arrow) are in the posterior tibial artery. The 2nd guidewire (GW) is indicated by the white arrow on the left side of the IVUS (right anterior oblique side). **D** IVUS findings of the chronic total occlusion lesion. The detector direction where the transducer and target plaque are maximally separated is at RAO4° (red arrow). Blue arrow: IVUS transducer; yellow arrow: IVUS wire (1st guidewire); white arrow: 2nd GW successfully advanced into the target plaque

AnteOwl WR (AnteOwl) (TERUMO, Tokyo, Japan) is a new intravascular ultrasound (IVUS) specifically developed for CTO intervention (Fig. 1 A, B) (Okamura et al. 2020). This IVUS makes it easy to project the directionality of the target plaque onto angiography by utilizing the asymmetric structure of the IVUS transducer and guidewire (Fig. 1 C, D). The maximum outer diameter of AnteOwl is 4.0 Fr, which allows its use with a typical 4.0 Fr or larger sheath.

Herein, we report the application of our IVUS-guided parallel wiring technique for BTK lesions and devised a new technique in which the guidewire is passed through the distal true lumen by repeatedly using this technique to advance into the true lumen. We describe this novel technique of extreme antegrade guidewire crossing by the AnteOwl IVUS-guided parallel wiring to a BTK artery (EXCAVATOR technique).

## Case presentations

### Case 1

**Fig. 2:**
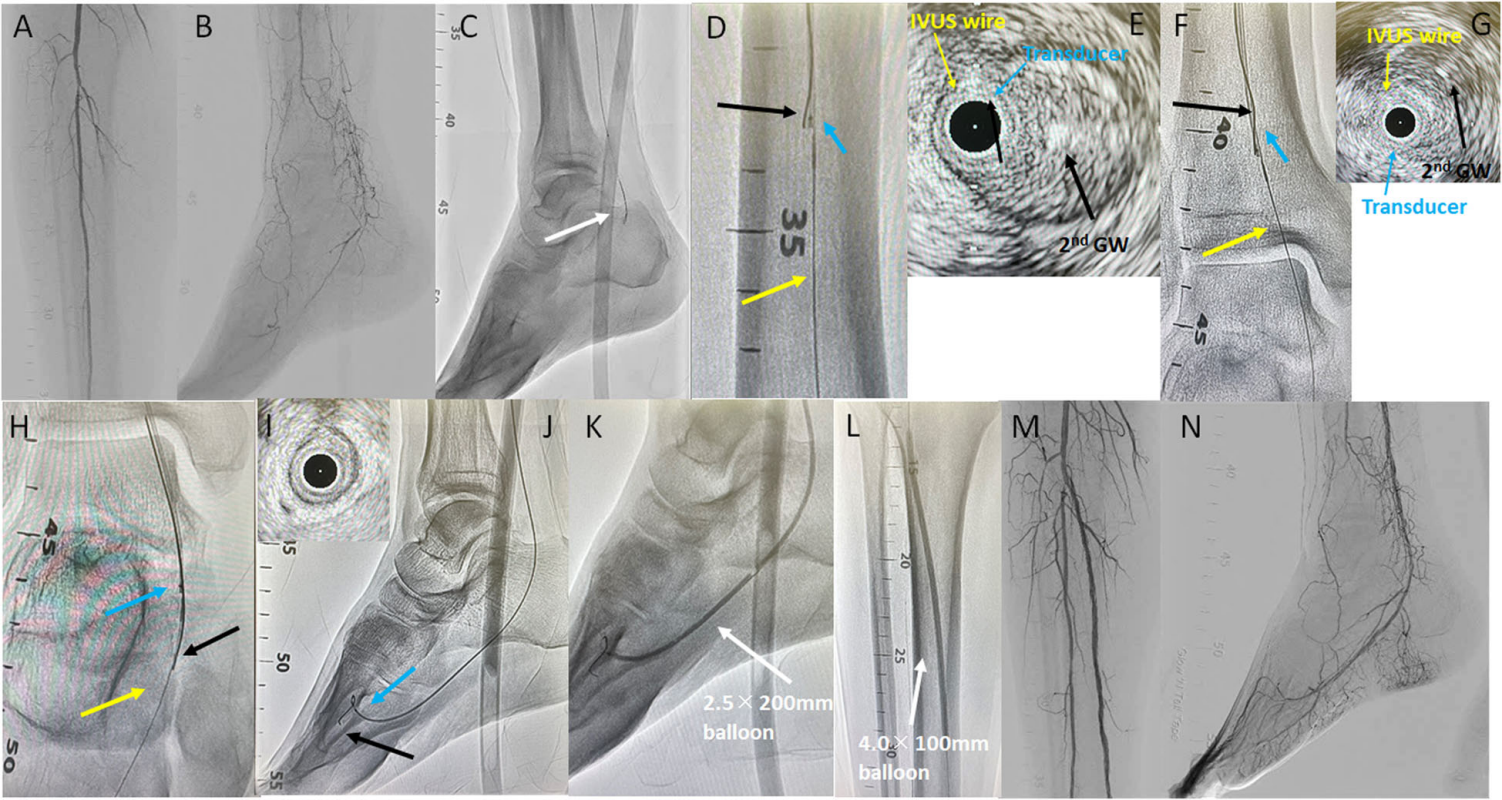
** A, B** Control angiography shows total occlusion of both tibial arteries, and very poor inframalleolar arteries. **C** A 0.014-inch guidewire (GW) is advanced into the subintimal space (white arrow: GW tip) in the posterior tibial artery (PTA). **D, F, H** Intravascular ultrasound (IVUS)-guided parallel wiring (**D**: proximal-to-middle PTA; **F**: distal PTA; **H**: lateral plantar artery). Yellow arrow: 1st GW and IVUS catheter; blue arrow: IVUS transducer; black arrow: 2nd GW advanced into the intraplaque of the PTA. **E, G** IVUS findings of the chronic total occlusion (CTO) lesion in the PTA (**E**: proximal-to-middle PTA; **G**: distal PTA). Yellow arrow: IVUS wire; blue arrow: IVUS transducer; black arrow: 2nd GW in the intraplaque route. **I** IVUS findings in the distal part of the lateral plantar artery. The IVUS transducer is almost in the center of the vessel. **J** The 2nd GW (black arrow) is advanced into the distal true lumen of the digital artery. Blue arrow: IVUS transducer. **K** A 2.5 × 200-mm balloon is dilated in the lateral plantar artery to the distal PTA. **L** A 4.0 × 100-mm balloon is dilated in the proximal PTA. **M, N** Final angiography shows sufficient dilation, antegrade blood flow, and small branches

A 70-year-old man presented with rest pain, cyanosis, intractable ulceration, and gangrene in the right toe. His ankle-brachial index (ABI) was 0.65 on the right side. The skin perfusion pressure was 21 mmHg dorsally and 10 mmHg at the plantar aspect. A 6-Fr guiding sheath (Destination® guiding sheath; TERUMO) was inserted into the right common femoral artery (CFA) via the ipsilateral antegrade approach. Control angiography showed total occlusion of the anterior tibial artery to the dorsalis pedis artery (DPA), and total occlusion of the posterior tibial artery (PTA) to the lateral plantar artery (PLA) (Fig. 2 A, B). A 0.014-inch guidewire (Gladius MGES® guidewire; Asahi Intec, Aichi, Japan) and a microcatheter (Caravel® microcatheter; Asahi Intec) seemed to be advanced into the subintimal space in the PTA CTO (Fig. 2 C). We advanced the AnteOwl into the CTO. IVUS showed that the guidewire was in the subintimal space from the middle part of the PTA CTO. We performed the IVUS-guided parallel wiring technique using the AnteOwl. We converted the direction of IVUS findings to angiography using the rotational angiography and asymmetrical structure of the IVUS transducer. We advanced a 0.014-inch guidewire (Halberd® guidewire; Asahi Intec) and microcatheter (Caravel® microcatheter; Asahi Intec) a few millimeters to the left (right anterior oblique side) of the IVUS on angiography, and advanced through the intraplaque route (Fig. 2D–H). Thus, we finally succeeded in passing the guidewire into the distal true lumen of the digital artery, and used IVUS to confirm that the true lumen was secured (Fig. 2I J). Based on the external elastic membrane-based vessel diameter accurately measured by IVUS, the PLA was dilated with a 2.5 × 200-mm balloon (SHIDEN HP®; Kaneka, Tokyo, Japan), the distal and middle PTA was dilated with a 3.0 × 200-mm balloon (SHIDEN HP®), and the proximal PTA was dilated with a 4.0 × 100-mm balloon (SHIDEN HP®) (Fig. 2 K, L). Final angiography showed sufficient expansion of the target lesion and good antegrade flow and blood flow in the side branches (Fig. 2 M, N). Procedural time of EVT was 100 min, the dose of radiation exposure used in terms of dose area product was 7.0 Gycm^2^, and the amount of contrast medium was 150 ml. Immediately after EVT, the cyanotic color of the skin on the right toe markedly improved. The postoperative ABI improved to 0.88. The wound on the right toe healed within 5 months. No additional EVT was required.

### Case 2

**Fig. 3:**
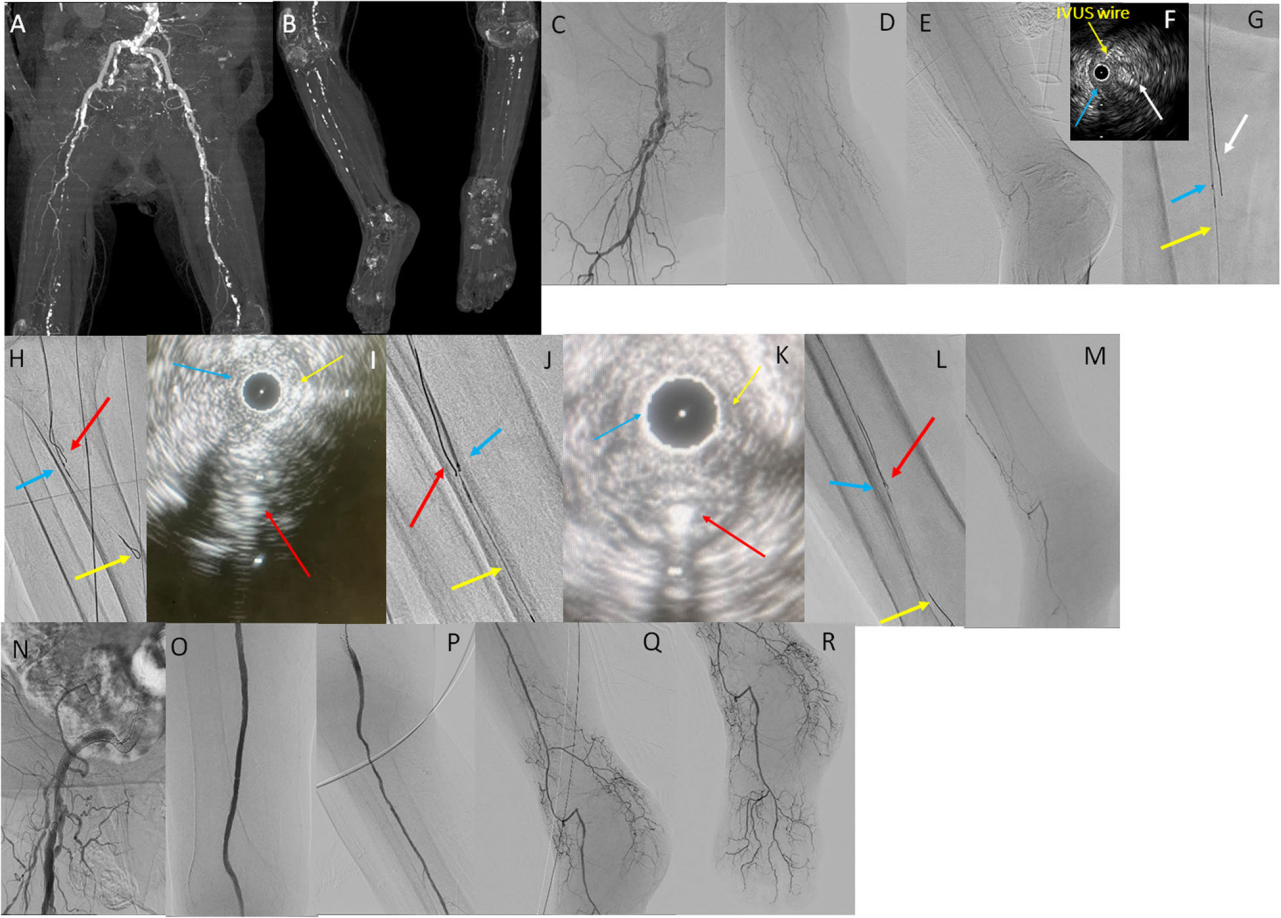
** A, B** Preprocedural enhanced computed tomography. **C, D, E** Control angiography shows total occlusion from the right superficial femoral artery (SFA) to below-the-knee arteries. Distal target vessels are very faint. **F** IVUS findings of the CTO lesion in the SFA. Yellow arrow: IVUS wire; blue arrow: IVUS transducer; white arrow: 2nd GW in the intraplaque route. **G** Intravascular ultrasound (IVUS)-guided parallel wiring (**G**: SFA). Yellow arrow: 1st guidewire (GW) and IVUS catheter; blue arrow: IVUS transducer; white arrow: 2nd GW advanced into the intraplaque of the chronic total occlusion (CTO) lesion. **H, J, L** IVUS-guided parallel wiring (**H**: distal popliteal artery to peroneal artery; **J**: peroneal artery; **L**: distal peroneal artery). Yellow arrow: 1st GW; blue arrow: IVUS transducer; red arrow: 2nd GW advanced into the intraplaque of the CTO lesion. **I, K** IVUS findings of the CTO lesion in the below-the-knee artery (**I**: distal popliteal artery to peroneal artery; **K**: peroneal artery). Yellow arrow: IVUS wire; blue arrow: IVUS transducer; red arrow: 2nd GW in the intraplaque. **M** Tip injection from the distal peroneal artery shows the distal true lumen of below-the-ankle arteries. **N, O, P, Q, R** Final angiography shows sufficient antegrade blood flow

A 76-year-old woman with diabetes mellitus presented with rest pain and cyanosis of both lower limbs. Her ABI could not be measured in either leg. Contrast-enhanced computed tomography showed total occlusion of the right SFA to the popliteal artery (PopA) to the BTK arteries (Fig. 3 A, B).

We inserted a 6-Fr guiding sheath (Crossroads® guiding sheath; NIPRO, Tokyo, Japan) from the left common femoral artery and used a contralateral crossover approach. Control angiography showed moderate stenosis of the distal EIA and total occlusion of the SFA from just proximal to the BTK arteries (Fig. 3 C-E). The distal DPA was barely imaged, while the main trunk of the tibial and peroneal arteries was not imaged at all. We advanced a 0.014-inch guidewire (Jupiter X; Boston Scientific) and microcatheter (Ichibanyari PAD2® microcatheter; Kaneka) to the CTO lesion in the SFA. When the AnteOwl IVUS was inserted at the proximal PopA, it was in the subintimal space from the mid-SFA, so IVUS-guided parallel wiring was started using a 0.014-inch guidewire (Astato XS9-40® guidewire; Asahi Intec) with a microcatheter (Ichibanyari PAD2® microcatheter; Kaneka) (Fig. 3 F, G). We advanced the guidewire along the intraplaque route and reached the distal PopA. However, the total occlusion continued to the BTK arteries. We again performed IVUS-guided parallel wiring using a 0.014-inch guidewire (Halberd® guidewire; Asahi Intec) with a microcatheter (Ichibanyari PAD2® microcatheter; Kaneka) (Fig. 3 H, I). By repeating these steps of IVUS-guided parallel wiring several times, we finally succeeded in passing the guidewire through the distal true lumen of the peroneal artery (Fig. 3 J-L). Tip injection confirmed that the distal true lumen of the peroneal artery was connected to the DPA via a collateral tract (Fig. 3 M). Appropriate vessel size evaluation and size of stent selection were performed by observation with IVUS, a 10.0 × 80-mm stent (Epic®; Boston Scientific) was deployed for the EIA, a 7.0 × 120-mm drug-eluting stent, 7.0 × 120-mm stent, and 6.0 × 120-mm stent (Eluvia®; Boston Scientific) were deployed for the SFA just proximal to the proximal PopA, and a 4.0 × 150-mm drug-coated balloon (Ranger®; Boston Scientific) was dilated in the distal PopA. However, there was a large dissection at the edge of the distal stent, and an additional 6.0 × 40-mm stent (Eluvia®; Boston Scientific) was deployed for the middle of the PopA. The peroneal artery was dilated with a 3.0 × 200-mm balloon (SHIDEN HP®; Kaneka). Final angiography showed very good antegrade blood flow to the periphery (Fig. 3 N-R). We treated EIA stenosis and continuous occlusion from the SFA to the peroneal artery with only a contralateral crossover antegrade approach. Procedural time for EVT was 278 min, the dose of radiation exposure was 60.6 Gycm^2^, and the amount of contrast medium was 175 ml. The postoperative ankle-brachial index improved to 0.9, and the clinical course was good.

## Discussion

We reported successful revascularization for long CTO lesions with very poor run-off target vessels using the EXCAVATOR technique, which brings the IVUS as far distal as possible to the BTK CTO and uses parallel wiring to allow antegrade guidewire passage to the distal true lumen under IVUS guidance. We previously reported the feasibility of EVT for FP CTO using the AnteOwl IVUS-guided approach and described the method used to perform AnteOwl IVUS-guided intraplaque wiring in FP CTO (Hayakawa et al. 2021). We converted IVUS images into angiographic images to navigate the second guidewire through an intraplaque route under IVUS observation from the subintimal space using the bias between asymmetrical structures of the IVUS transducer and IVUS guidewire. Using this technique, the target true lumen on either side of the IVUS catheter could be converted on the angiographic image, and if the guidewire was advanced in that direction, the guidewire could pass through the true lumen. However, to the best of our knowledge, this is the first report of the use of AnteOwl IVUS in BTK CTO intervention.

The primary strategy for BTK CTO is generally to use antegrade wiring; however, a previous clinical studies reported a relatively high rate of antegrade guidewire crossing failure (Kokkinidis et al. 2020; Tan et al. 2021). Various retrograde approaches have improved the success rate of the procedure in BTK CTO; however, lesions with poor distal target vessels are extremely difficult to treat with retrograde approaches (Schmidt et al. 2017). In the two cases experienced, distal target vessels were scarce, making it difficult to use the retrograde approach.

To overcome these issues associated with treating complex BTK CTO lesions, we devised a novel antegrade AnteOwl IVUS-guided wiring technique called the EXCAVATOR. AnteOwl is a novel IVUS with an asymmetrical structure of the proximal transducer and IVUS wire, facilitating the reflection of IVUS findings onto angiographic images to allow accurate navigation of the second guidewire into the intraplaque route. AnteOwl has a small profile and crossable catheter, durable hydrophilic coating, and high-resolution imaging.

**Fig. 4:**
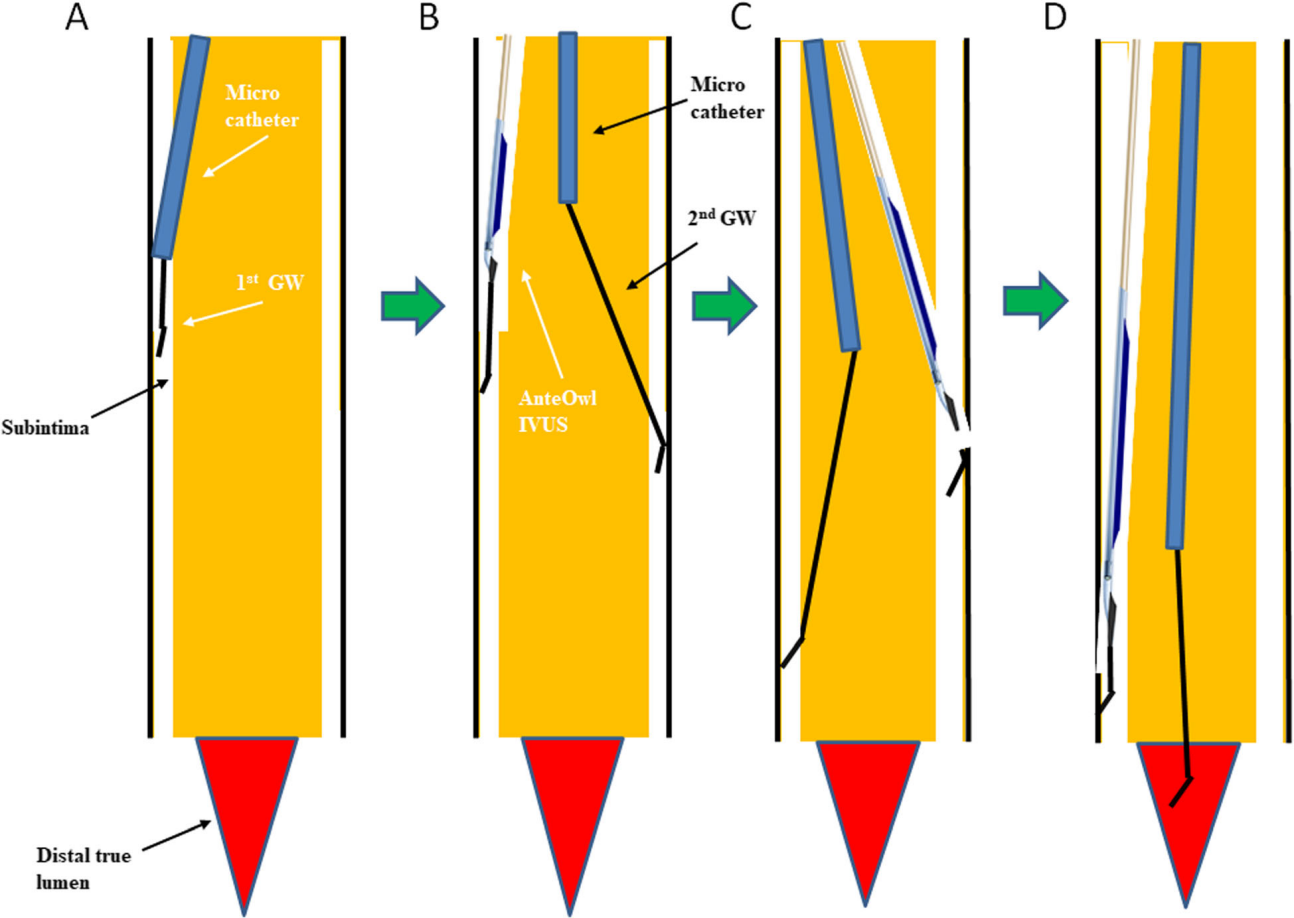
Schematic diagram of extreme antegrade guidewire crossing by AnteOwl WR intravascular ultrasound (IVUS)-guided parallel wiring to a below-the-knee artery (EXCAVATOR). **A** The 1st guidewire (GW) with microcatheter is advanced into the subintima. We use the bougie technique with the microcatheter to prepare for IVUS insertion. **B** The AnteOwl IVUS is advanced along the 1st GW. The 2nd GW with microcatheter is inserted to perform IVUS-guided parallel wiring. The 2nd GW is guided to the intraplaque under real-time IVUS guidance. The 2nd GW is then advanced into the subintima. **C** The bougie technique is used as shown in (**A)** and the IVUS is returned to the subintima. Under IVUS guidance, the 2nd GW is again introduced into the plaque. **D** Finally, the 2nd GW is passed from the distal plaque to the distal true lumen

The steps of IVUS-guided parallel wiring are as follows. The guidewire is first advanced as far distally as possible in an antegrade fashion. Next, we advance the microcatheter as far distally as possible and use the bougie effect, a method to dilate a stenosis by passing a microcatheter through, to insert the AnteOwl (Fig. 4 A). When the first guidewire is in the subintimal space, we manipulate the second guidewire into the intraplaque route under IVUS guidance (Fig. 4B). The IVUS catheter can be observed up to the point where it has been advanced within the CTO, and a second guidewire can be advanced to an intraplaque route by IVUS-guided parallel wiring. When the guidewire is advanced to the area that cannot be observed by IVUS, we step down to a floppy or intermediate type of guidewire and advance this guidewire under angiographic guidance. By repeating this process and proceeding along the intraplaque route, the guidewire can finally be passed through the distal lumen (Fig. 4 C, D). This IVUS-guided parallel wiring allows the passage of all intraluminal guidewires. The first guidewire is advanced as far as possible distal to the CTO, even if it is subintimal, and eventually the second guidewire is advanced into the true lumen guided by IVUS.

In recent years, IVUS has been used to determine the appropriate vessel diameter for BTK lesions, and clinically, it has accelerated improvements in wound care (Fujihara et al. 2020; Soga et al. 2021). In addition to the evaluation of the appropriate vessel diameter, the AnteOwl may also allow guidewire passage in difficult cases and reduce the incidence of distal puncture.

This AnteOwl-guided guidewire crossing has limitations because severe calcification may not enable the passage of the IVUS, or it may be poorly observed by IVUS. The presence of severe calcification makes it difficult to see on IVUS and prevents penetration of the guidewire. Case 2 was considerably more difficult than case 1, in part because of the presence of severe calcification, which required longer procedural time. Another limitation is that it is an antegrade intraluminal approach, which may require longer procedural time. In both cases, the retrograde approach was difficult to perform, especially in Case 2, where a very long occlusion from the SFA to the distal part of the peroneal artery was treated in a single term, but which still required a very long procedure time. Longer procedure time means increased radiation exposure for the patient and the operator. Case 2 required a longer procedure time and higher radiation dose than Case 1. Although these values were acceptable when compared with a previous report of the radiation dose for coronary angiography and intervention (Nakamura et al. 2016), the increased procedure time and radiation dose should be noted. In cases where a retrograde approach can be performed easily, we advise shortening the procedure time by shifting to a retrograde approach as soon as possible. A much larger study is required to confirm the efficacy of AnteOwl IVUS-guided EVT for BTK CTO.

## Conclusions

AnteOwl IVUS was effective for antegrade guidewire crossing to treat BTK CTO. The EXCAVATOR technique can be used to treat long BTK CTO with poor target vessels.

## Data Availability

The datasets used and/or analyzed during the current study are available from the corresponding author on reasonable request.

## Abbreviations

CTO: Chronic total occlusion
BTK: Below-the-knee
IVUS: Intravascular ultrasound
EXCAVATOR: Extreme antegrade guidewire crossing by AnteOwl WR intravascular ultrasound-guided parallel wiring to a BTK artery
PTA: Posterior tibial artery
SFA: Superficial femoral artery
EVT: Endovascular therapy
CLTI: Chronic limb-threatening ischemia
FP: Femoropopliteal
pDVA: Percutaneous deep venous arterialization
EIA: External iliac artery
DPA: Dorsalis pedis artery
PLA: Plantar artery
PopA: Popliteal artery
CFA: Common femoral artery
